# Novel *TTC37* Mutations in a Patient with Immunodeficiency without Diarrhea: Extending the Phenotype of Trichohepatoenteric Syndrome

**DOI:** 10.3389/fped.2015.00002

**Published:** 2015-01-30

**Authors:** Nicholas L. Rider, Bertrand Boisson, Soma Jyonouchi, Eric P. Hanson, Sergio D. Rosenzweig, Jean-Laurent Casanova, Jordan S. Orange

**Affiliations:** ^1^Department of Immunology, Allergy and Rheumatology, Baylor College of Medicine, Texas Children’s Hospital, Houston, TX, USA; ^2^St. Giles Laboratory of Human Genetics of Infectious Diseases, Rockefeller University, New York, NY, USA; ^3^Department of Allergy-Immunology, The Children’s Hospital of Philadelphia, Philadelphia, PA, USA; ^4^Immunodeficiency and Inflammation Unit, National Institute of Arthritis, Musculoskeletal and Skin Disease, National Institutes of Health, Bethesda, MD, USA; ^5^Laboratory of Host Defense, National Institute of Allergy and Infectious Disease, National Institutes of Health, Bethesda, MD, USA; ^6^Necker Hospital for Sick Children, Imagine Institute, INSERM, HHMI, Paris Descartes University, Paris, France; ^7^Howard Hughes Medical Institute, New York, NY, USA

**Keywords:** trichohepatoenteric syndrome, primary immunodeficiency, ectodermal dysplasia, trichorrhexis nodosa, antibody deficiency, chronic diarrhea

## Abstract

Unbiased genetic diagnosis has increasingly associated seemingly unrelated somatic and immunological phenotypes. We report a male infant who presented within the first year of life with physical growth impairment, feeding difficulties, hyperemesis without diarrhea, and abnormal hair findings suggestive of trichorrhexis nodosa. With advancing age, moderate global developmental delay, susceptibility to frequent viral illnesses, otitis media, and purulent conjunctivitis were identified. Because of the repeated infections, an immunological evaluation was pursued and identified impaired antibody memory responses following pneumococcal vaccine administration. Immunoglobulin replacement therapy and nutritional support were employed as mainstays of therapy. The child is now aged 12 years and still without diarrhea. Whole exome sequencing identified compound heterozygous mutations in the *TTC37* gene, a known cause of the trichohepatoenteric syndrome (THES). This case extends the known phenotype of THES and defines a potential subset for inclusion as an immune overlap syndrome.

## Introduction

Trichohepatoenteric Syndrome (THES) is a rare autosomal recessive disorder characterized by growth restriction, severe infantile diarrhea, trichorrhexis nodosa-like hair morphology, hepatopathy, facial dysmorphism, and immunodeficiency (1–5). While THES1 (MIM 222470) is caused by mutation of the *TTC37* gene, THES2 (MIM 614602) results from *SKIV2L* gene mutations (6–8). Both genes encode members of the human Ski complex, which functions in exosomal RNA degradation (8). The clinical features of patients with THES1 are indistinguishable from those with THES2; thus, THES is considered a single entity with two molecular etiologies (2).

A recent report of 15 patients with THES treated similarly and followed longitudinally showed that 5 year survival was 86.7% (9). Three (20%) patients died of infectious complications. All 15 patients required total parenteral nutrition for intractable diarrhea and 9 (60%) required immunoglobulin infusions for variable duration but 10 (67%) patients were found to have detectable humoral immune defects (9). Two (13%) of the patients had an inflammatory colitis and nine (60%) had transient hemophagocytosis. Other THES patients are reported to have immunologically relevant characteristics including: abnormal T-cell response to *in vitro* stimulation; persistent hypogammaglobulinemia; transient hypogammaglobulinemia; specific antibody deficiency; fatal measles infection; and inflammatory colitis (6, 8, 10, 11). At present, the immunological phenotype of THES is neither well characterized nor understood, but appears to be a significant feature of the syndrome.

## Background

**Figure 1:**
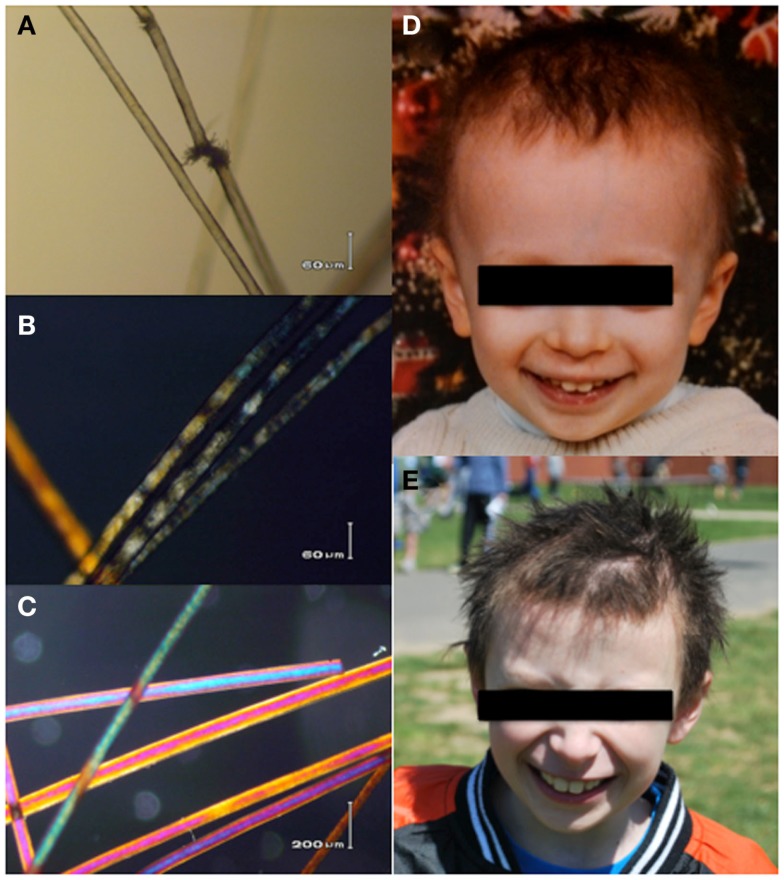
**The patients hair shafts have trichorrhexis nodosa-like defects (A), and under polarizing microscopy (B) show a somewhat irregular banding pattern reminiscent of, but distinct from the tiger tail banding characteristic of trichothiodystrophy**. These are not seen in the polarized view of hair shafts from his brother **(C)**. Pastel colors are artifacts. **(D,E)** Show the patients facial features and gross hair morphology at ages 2 and 10 years, respectively. Hair analysis and photos compliments of Alan Zhou, John J. DiGiovanna, Deborah Tamura, and Kenneth H. Kraemer. Patient photos courtesy of the family archive, used with permission.

A Caucasian male infant was born at 36 weeks gestation to unrelated parents. His birth length was at the 10th percentile but weight was <3rd percentile. Early in the first year of life, somatic growth impairment was evident (weight and length ≤3rd %) and associated with feeding intolerance. His hair was fine, sparse, and brittle with a wooly character; facial features were striking (Figure 1). Diarrhea was occasional, but only intermittent and mild. Vomiting, however, was persistent, often debilitating and associated with aphthous oral ulcers. He required placement of a gastrostomy tube at 22 months of age and was hospitalized for implementation of a feeding regimen shortly thereafter. Gastrointestinal evaluation including endoscopy was grossly normal.

**Figure 2:**
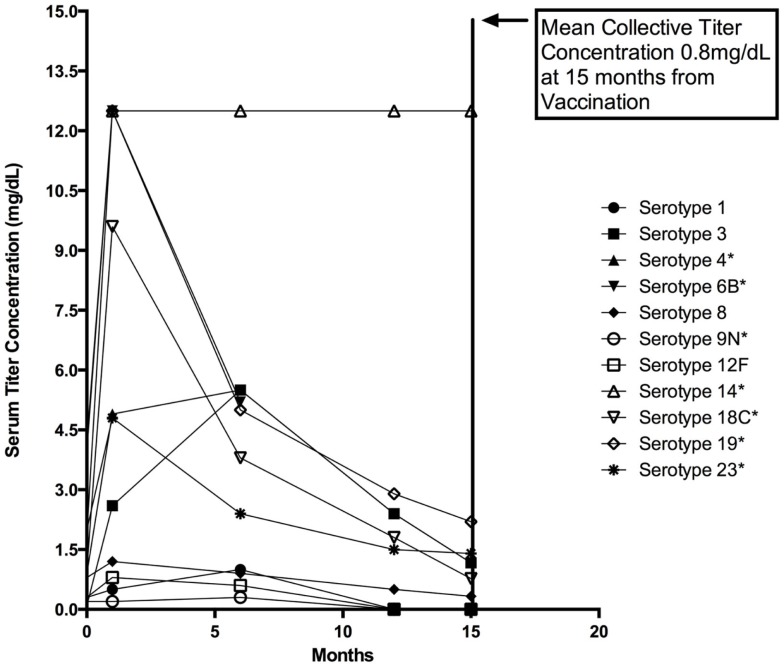
**Twenty-three valent pneumococcal vaccine response**. Various pneumococcal vaccine serotype titers plotted together before vaccination and at represented intervals following vaccination with Pneumovax © at 4, 24, 52, and 60 weeks. Vaccination was performed at 3 years of age following a complete series of the pneumococcal conjugate (Prevnar 7©) vaccine at recommended ages (AAP schedule) (13). Note the serotypes indicated with an asterisk (*) indicate those included in the Prevnar 7 vaccine. For reference, minimally protective titer levels are accepted to be ≥1.3 mg/dL [Bonilla et al. (14)]. Mean and one standard deviation are represented for each group. Mean serotype titers for each time point shown are: 1.4 μg/dL (Pre), 3.1 μg/dL (4 weeks), 2.4 μg/dL (24 weeks), 1.0 μg/dL (52 weeks), and 0.80 g/dL (60 weeks).

The patient experienced frequent otitis media, viral infections, and purulent conjunctivitis despite trimethoprim–sulfamethoxazole prophylaxis. Simple upper respiratory tract infections often progressed to presumably bacterial pulmonary infections requiring additional antimicrobial therapy. Immunological evaluation of cell counts, lymphocyte proliferation to mitogens, Toll-like receptor function, and serum quantitative immunoglobulin concentrations were normal; however, response to pneumococcal vaccination was abnormal with rapid loss of protective titers (Figure 2) (12). Immunoglobulin replacement therapy was initiated at 4 years of age owing to recurring bacterial infections despite continual antibiotic prophylaxis and rapid pneumococcal-specific antibody titer decline. The patient had no history of invasive or opportunistic infections and hemophagocytosis was absent. Microscopy of our patient’s hair showed trichorrhexis nodosa-like findings similar to those found in other patients reported with THES, which were (Figure 1) remarkably different from that of his parents and sibling (9).

**Figure 3:**
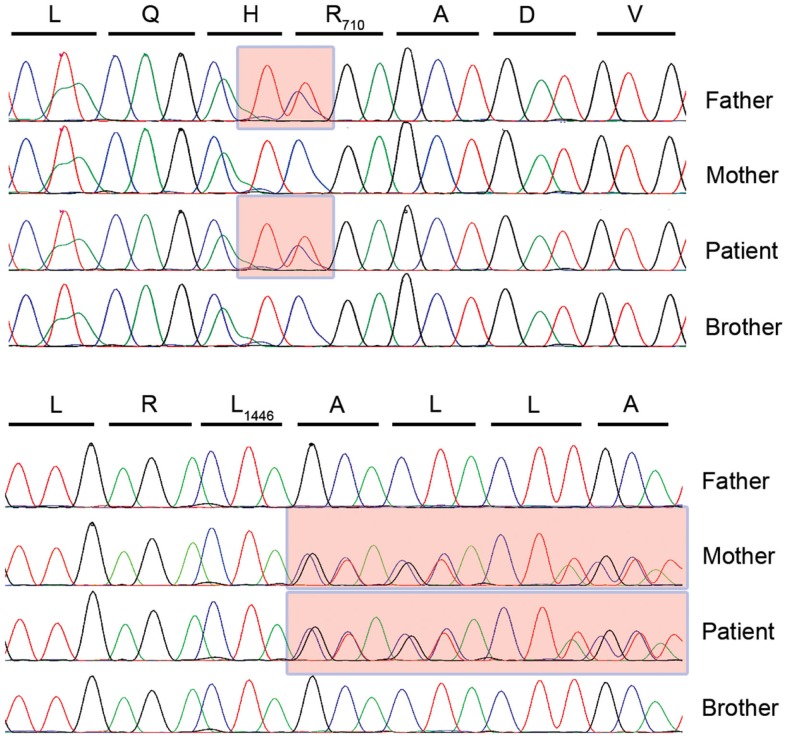
**TTC37 sequence electropherograms for the four members of the kindred in the region corresponding to the paternally inherited c. 2128C > T mutation (upper panel) and the maternally inherited c. 4337_4338insCTA insertion (lower panel)**.

In the absence of a clear clinical diagnosis and following acquisition of parental consent, whole exome sequencing was completed using DNA from ficoll pellet from P1 and his brother (15). After filtering for rare or non-reported variant, compound heterozygous variants in the *TTC37* gene were found (c. 2128C > T and c. 4337_4338insCTA) in the patient leading to a stop in position 710 (R710X) and an in-frame Leu insertion in position 1446 (L1446_A1447insL), respectively suggesting a diagnosis of THES. Each parent carried one mutation and the healthy sibling had no defects in the *TTC37* gene (Figure 3). The patient subsequently had a liver ultrasound and hepatic function testing, which were unremarkable.

Presently at 12 years of age, our patient suffers from recurrent aphthous ulcers and purulent conjunctivitis without a clear microbiological cause. He is small for age but vigorous and of normal intelligence. He remains on weekly subcutaneous immunoglobulin therapy, which is well tolerated. A reduction in the need for antibiotics and more robust resistance to infection were noted since initiating immunoglobulin replacement therapy.

## Discussion

Trichohepatoenteric Syndrome is a rare autosomal recessive disorder with hallmark clinical findings of growth impairment, severe diarrhea, hair abnormalities, and immune deficiency (2, 6, 8, 9). We extend the spectrum of the immunological deficiency associated with THES in our patient to include specific antibody deficiency with impairment of humoral memory. Our patient also extends the somatic phenotype attributed to *TTC37* mutation as he did not have the typical severe diarrhea of infancy characteristic of THES, despite having the typical hair features. His liver function and morphology is also normal to this point. Genotype–phenotype correlations for THES are not well understood; however, our patient’s lack of severe gastrointestinal symptoms may portend a better prognosis across the THES spectrum despite immunodeficiency. At 12 years of age, he will require ongoing monitoring for the development of gastrointestinal and other systemic symptoms as knowledge regarding the natural history of THES through adolescence and adulthood is lacking.

The detailed immunological evaluation of our patient suggests that global innate immunity is normal in THES patients. His normal cell counts and Toll-like receptor function are potentially relevant to the evaluation of other THES patients and while they do not exclude the diagnosis these features may help differentiate THES from ectodermal dysplasia with immunodeficiency and related disorders. Our patient’s abnormal response to pneumococcal vaccination is reflective of humoral immune defects in other patients with THES and may suggest a role for *TTC37* in adaptive immune function. The mechanism of defective humoral memory is not clear in this case as our patient displayed normal B-memory cell (CD19+27+) enumeration on several occasions and had an initial response to vaccination with subsequent rapid decline in protective titers (Figure 2). Further consideration of this phenotype specifically in patients with THES will hopefully prove informative. Consideration of rapid loss of protective antibodies can easily be overlooked in immunological evaluations and is another important lesson to be learned from this case.

## Concluding Remarks

In summary, we present a 12-year-old male with novel compound heterozygous variants in *TTC37*, abnormal antibody production with growth and hair abnormalities who does not display typical THES-related gastrointestinal features. This case underscores the importance of a thorough immunological evaluation and antibody replacement therapy given the relatively high prevalence of immunodeficiency in THES patients (3, 4, 6, 9). Additionally, this is the first report of a patient from the United States with THES, thereby expanding the global spectrum of this disease. Finally, it illustrates the power of unbiased whole exome sequencing for the diagnosis of novel clinical presentations of known inborn errors (15).

## Conflict of Interest Statement

The authors declare that the research was conducted in the absence of any commercial or financial relationships that could be construed as a potential conflict of interest.
