# Corrigendum: Novel TTC37 Mutations in a Patient with Immunodeficiency without Diarrhea: Extending the Phenotype of Trichohepatoenteric Syndrome

**DOI:** 10.3389/fped.2015.00028

**Published:** 2015-04-16

**Authors:** Nicholas L. Rider, Bertrand Boisson, Soma Jyonouchi, Eric P. Hanson, Sergio D. Rosenzweig, Jean-Laurent Casanova, Jordan S. Orange

**Affiliations:** ^1^Department of Immunology, Allergy and Rheumatology, Baylor College of Medicine, Texas Children’s Hospital, Houston, TX, USA; ^2^St. Giles Laboratory of Human Genetics of Infectious Diseases, Rockefeller University, New York, NY, USA; ^3^Department of Allergy-Immunology, The Children’s Hospital of Philadelphia, Philadelphia, PA, USA; ^4^Immunodeficiency and Inflammation Unit, National Institute of Arthritis, Musculoskeletal and Skin Disease, National Institutes of Health, Bethesda, MD, USA; ^5^Laboratory of Host Defense, National Institute of Allergy and Infectious Disease, National Institutes of Health, Bethesda, MD, USA; ^6^Necker Hospital for Sick Children, Imagine Institute, INSERM, HHMI, Paris Descartes University, Paris, France; ^7^Howard Hughes Medical Institute, New York, NY, USA

**Keywords:** trichohepatoenteric syndrome, primary immunodeficiency, ectodermal dysplasia, trichorrhexis nodosa, antibody deficiency, chronic diarrhea

During the preparation of the paper the authors misspelled the name of author Jean-Laurent Casanova as Jean-Laurent Cassanova. The author list should read:

Nicholas L. Rider, Bertrand Boisson, Soma Jyonouchi, Eric P. Hanson, Sergio D. Rosenzweig, Jean-Laurent Casanova and Jordan S. Orange

The authors would like to apologize for any potential inconvenience caused.

The original article has been updated.

## Conflict of Interest Statement

The authors declare that the research was conducted in the absence of any commercial or financial relationships that could be construed as a potential conflict of interest.

